# Deficient Sleep in Mouse Models of Fragile X Syndrome

**DOI:** 10.3389/fnmol.2017.00280

**Published:** 2017-09-01

**Authors:** R. Michelle Saré, Lee Harkless, Merlin Levine, Anita Torossian, Carrie A. Sheeler, Carolyn B. Smith

**Affiliations:** Section on Neuroadaptation and Protein Metabolism, Department of Health and Human Services, National Institute of Mental Health (NIMH), National Institutes of Health (NIH) Bethesda, MD, United States

**Keywords:** sleep disruption, home-cage monitoring system, Fragile X, *Fmr1*, *Fxr2*

## Abstract

In patients with fragile X syndrome (FXS), sleep problems are commonly observed but are not well characterized. In animal models of FXS (*dfmr1* and *Fmr1* knockout (KO)/*Fxr2* heterozygote) circadian rhythmicity is affected, but sleep *per se* has not been examined. We used a home-cage monitoring system to assess total sleep time in both light and dark phases in *Fmr1* KO mice at different developmental stages. *Fmr1* KOs at P21 do not differ from controls, but genotype × phase interactions in both adult (P70 and P180) groups are statistically significant indicating that sleep in *Fmr1* KOs is reduced selectively in the light phase compared to controls. Our results show the emergence of abnormal sleep in *Fmr1* KOs during the later stages of brain maturation. Treatment of adult *Fmr1* KO mice with a GABA_B_ agonist, R-baclofen, did not restore sleep duration in the light phase. In adult (P70) *Fmr1* KO*/Fxr2* heterozygote animals, total sleep time was further reduced, once again in the light phase. Our data highlight the importance of the fragile X genes (*Fmr1* and *Fxr2*) in sleep physiology and confirm the utility of these mouse models in enhancing our understanding of sleep disorders in FXS.

## Introduction

Fragile X syndrome (FXS) is an X-linked disorder caused by a CGG repeat expansion in the 5′UTR of *FMR1* resulting in gene silencing. FXS is a major inherited cause of intellectual disability and is also associated with autistic-like behaviors. Sleep abnormalities are a common symptom reported in patients with FXS (Picchioni et al., 2014) and are correlated with the severity of the behavioral phenotypes of the disorder (Kronk et al., 2010). The exact nature of sleep abnormalities is not well understood. The studies reporting sleep characteristics in FXS patients are confounded by the large age range of subjects studied, the variety of methods by which sleep is assessed, and medications used in the patients studied (Musumeci et al., 1995; Gould et al., 2000; Miano et al., 2008; Kronk et al., 2010).

Animal models of FXS provide a system in which many of the confounds of clinical studies can be avoided. Moreover, preclinical studies in animal models are critical to the investigation of efficacy of pharmacological interventions. To date, studies in animal models have focused more on circadian rhythm abnormalities than sleep deficiencies. In *Drosophila* models of FXS (*dfmr1*), an absence of circadian rhythm has been demonstrated (Dockendorff et al., 2002; Inoue et al., 2002; Sekine et al., 2008). In addition, two studies of the *dmfr1* model have shown that sleep is also dysregulated (Bushey et al., 2009; van Alphen et al., 2013). In mice, *Fmr1* deletion alone (*Fmr1* knockout, KO) results in a slightly shorter period length measured in free running mice housed in constant darkness (Zhang et al., 2008). In contrast to *Drosophila*, mammals also express *Fmr1* paralogs, *Fxr1* and *Fxr2*; both paralogs can interact with *Fmr1* (Zhang et al., 1995). Mice with mutations in both *Fmr1* and *Fxr2* have exaggerated behavioral phenotypes (Spencer et al., 2006) and a loss of circadian rhythm (Zhang et al., 2008). Sleep in these mice, however, has not been characterized.

Here, we report results of our studies of sleep in *Fmr1* KO mice. We studied mice at three ages, P21, P70 and P180, to determine the developmental course of sleep deficiencies. We also examined sleep in *Fmr1* KO*/Fxr2* Heterozygous (Het) animals at P70 to determine if the phenotype was made worse by the addition of an *Fxr2* mutation. We assessed sleep by means of a non-invasive home-cage monitoring-based system (Pack et al., 2007). Our results suggest that sleep disturbances increase over the lifecycle of *Fmr1* KO mice. At weaning (P21), total sleep time was not affected. At P70, *Fmr1* KO mice had reduced sleep in the light phase compared to controls. This phenotype persisted at P180 and was not rescued by treatment with a GABA_B_ agonist, R-baclofen. Additionally, P70 *Fmr1* KO*/Fxr2* Het animals had a further decrease in sleep in the light phase compared to *Fmr1* single mutants. These findings highlight the utility of *Fmr1* KO mice to understand sleep in FXS.

## Materials and Methods

### Animals

All mice were group housed in a standard housing environment with up to five mice per cage (except during sleep analysis) in a climate-controlled central facility with a 12:12 h (6:00 AM–6:00 PM) light:dark environment. Food and water were available to mice *ad libitum*. All procedures were carried out in accordance with the National Institutes of Health Guidelines on the Care and Use of Animals and approved by the National Institute of Mental Health Animal Care and Use Committee.

### *Fmr1* KO Breeding

These studies were conducted on male *Fmr1* hemizygous KO animals (*Fmr1* KO) and control littermates (on a C57BL/6J background), generated in house through Het female and WT male breeding pairs. Genotyping of mouse tail DNA by PCR amplification was previously described (Qin et al., 2002). In separate groups of animals, studies were initiated at 20–22 days of age (P21), 60–80 days of age (P70), or 170–190 days of age (P180).

### *Fmr1* KO/*Fxr2* Het Breeding

These studies were conducted on male *Fmr1* hemizygous KO animals, *Fxr2*^+/+^ (*Fmr1* KO/*Fxr2* WT) and *Fmr1* hemizygous KO animals, *Fxr2*^+/−^ (*Fmr1* KO/*Fxr2* Het) on a C57BL/6J background. Studies were conducted at 60–80 days of age. These mice were generated from female *Fmr1*^−/−^, Fxr2^+/−^ and male *Fmr1* hemizygous, *Fxr2*^+/−^ breeder pairs kindly provided by David Nelson (Baylor College of Medicine). The following primers were used to genotype *Fxr2*: (1) 5′-GTG ACA GTT TCC TGC TTT ACA GTC C; (2) 5′-TCT GCC TGC TTC CTG AGT GTT G; and (3) 5′-CGC CTT CTA TCG CCT TCT TGA C. Cycling conditions were as follows: 94°C for 5 min, (94°C for 60 s 54°C for 30 s and 72°C for 45 s) × 30 cycles, and 72°C for 7 min.

### Home-Cage Assessment of Sleep

Sleep was assessed by home-cage activity monitoring. Mice were singly housed in a clean standard cage surrounded by a rectangular arena of oppositely positioned infrared emitters and sensors (Comprehensive Laboratory Animal Monitoring System (CLAMS); Columbus Instruments, Columbus, OH, USA). Photobeams were spaced 0.5 inches apart on the x and y planes to assess activity on a high-resolution grid. The CLAMS software discriminated between fine movements (multiple breaks of the same beam) and locomotor activity (breaking two adjacent beams). For the analysis, the sums of fine and locomotor activities were used. The CLAMS software detected beam breaks in 10 s epochs. A mouse was considered inactive if there was no xy movement over the 10 s epoch, and 40 s of such inactivity was recorded as sleep. Validation of these measures as indicators of sleep in C57BL/6J mice was reported previously (Pack et al., 2007). The total amount of time asleep was separated into light phase (time asleep between 6:00 AM and 6:00 PM) and dark phase (time asleep between 6:00 PM and 6:00 AM) and then recorded as a percentage of the 12 h total time per phase. Sleep was analyzed for each 24 h period. For *Fmr1* KO mice, sleep was analyzed for six consecutive days. For *Fmr1* KO*/Fxr2* Het mice, sleep was analyzed for three consecutive days.

### R-Baclofen Treatment

In a separate group of animals, we used 18 *Fmr1* KO animals at 6 months of age to assess sleep duration prior to and during R-baclofen treatment. Mice were given saline injections, i.p., at 6:00 AM for 9 days. Sleep was assessed during the last 4 days of saline injections. R-baclofen was obtained from Seaside Therapeutics (Cambridge, MA, USA), dissolved in saline, and administered at 1.5 mg/kg i.p. at 6:00 AM for 2 days following the 9 days of saline injections (Days 10–11). The average sleep durations in the light and dark phases during saline injections were compared (Days 7–9) with sleep durations during R-baclofen injections (Days 10–11).

### Statistical Analysis

Data were analyzed by means of a mixed model repeated measures (RM) ANOVA. The between subjects’ variable was genotype. The within subjects’ variables were day and phase (light, dark). A criterion of *p* ≤ 0.05 was considered to be statistically significant. These results are indicated with an “*”. We compared sleep duration during saline injections with sleep duration during R-baclofen injections by means of a paired *t*-test.

## Results

### Habituation to Home-Cage Monitoring

**Figure 1:**
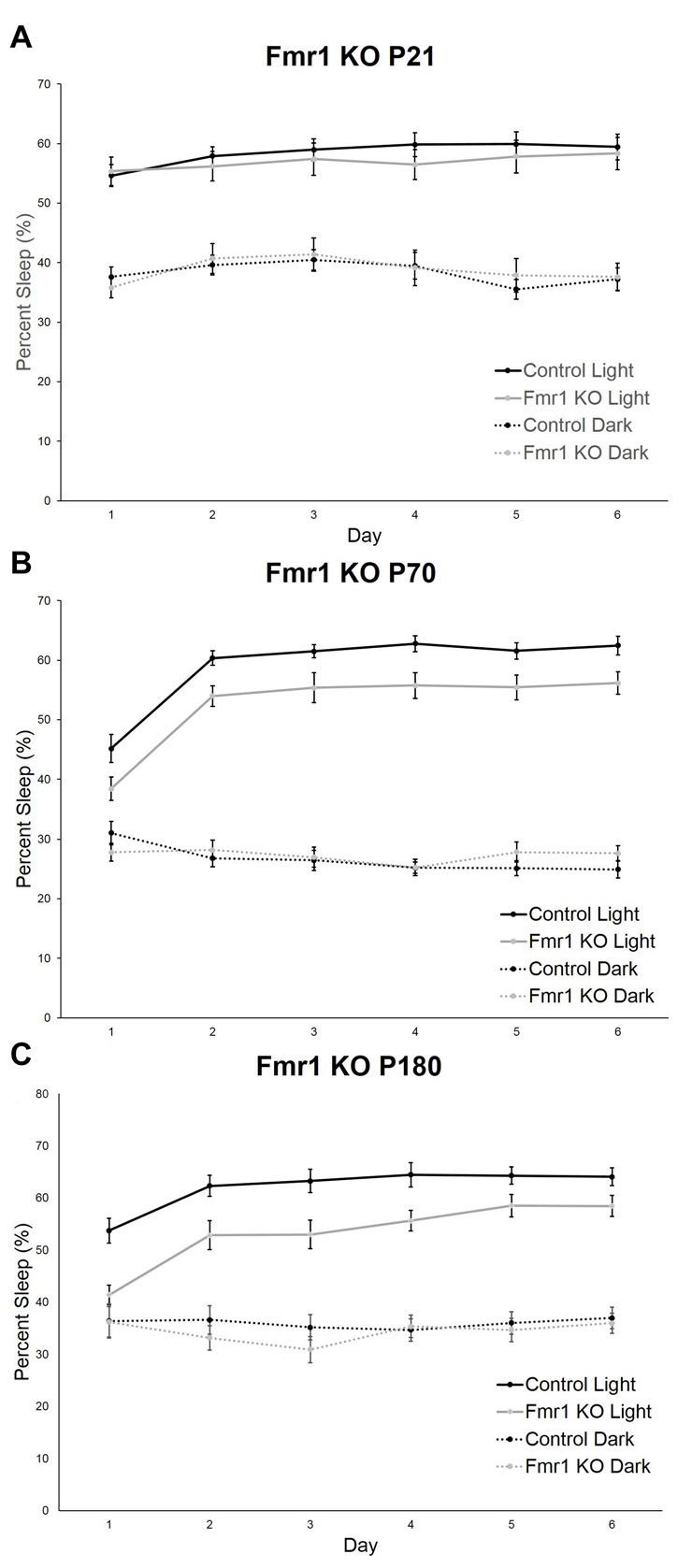
Habituation effect in control and *Fmr1* knockout (KO) mice across the 6-day testing period in the light and dark phases. Points are the means ± standard error of the mean (SEM). **(A)** At P21, there were no differences in genotype or in day in either phase. **(B)** At P70, the day × phase interaction was statistically significant (*p* < 0.001). *Post hoc t*-tests indicate that Day 1 differed from all other days in the light phase only. This habituation was not affected by genotype. **(C)** At P180, the day × phase interaction was statistically significant (*p* < 0.001). *Post hoc t*-tests indicate that Day 1 differed from all other days in the light phase only. This was not affected by genotype.

Recording commenced as soon as animals were housed in monitoring cages and continued for 6 days. We assessed the effect of day (habituation effect) on sleep in control and *Fmr1* KO mice at P21, P70 and P180. At P21, neither the day × phase nor the genotype × phase interaction was statistically significant. Furthermore, neither the main effect of day nor genotype was statistically significant. The main effect of phase was statistically significant. As expected in nocturnal animals, percent sleep time was longer in the light phase. Sleep was stable across the 6-day recording period and did not differ by genotype (Figure 1A). At P70, the day × phase interaction was statistically significant (*p* < 0.001). *Post hoc t*-tests revealed that sleep duration on Day 1 differed from Days 2–6 in the light phase only. The genotype × phase interaction was also statistically significant (*p* = 0.002; Figure 1B), indicating that *Fmr1* KO mice had a shorter sleep duration in the light phase. At P180, the day × phase interaction was statistically significant (*p* < 0.001). *Post hoc t*-tests revealed that Day 1 was different from all other days only in the light phase. The genotype × phase interaction was also statistically significant (*p* = 0.004; Figure 1C), indicating that the *Fmr1* KO mice had a shorter sleep duration in the light phase. These results show that habituation to the housing condition occurred during the first 24 h period, particularly in the adult animals, and that habituation was similar in both control and *Fmr1* KO mice.

### Developmental Course of Sleep Deficiencies in *Fmr1* KO Mice

The genotype × phase interaction in P70 and P180 animals indicates that there are differences between the genotypes in sleep time that depend on phase. To eliminate the effect of habituation, we confirmed these effects by analyzing the genotype × phase effects on average sleep time over Days 2–6.

**Figure 2:**
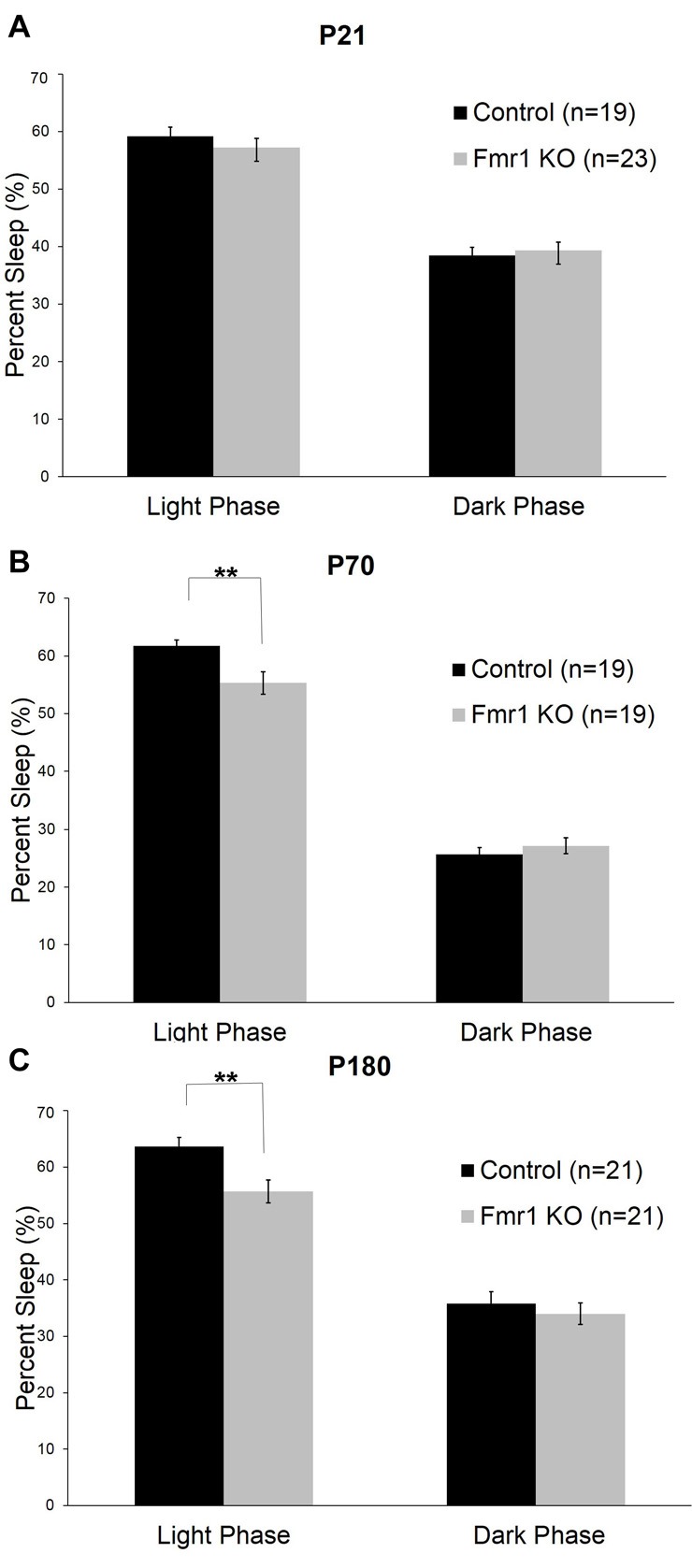
Sleep duration in control and *Fmr1* KO mice in light and dark phases. Bars are the means ± SEM of sleep averaged across Days 2–6 of the number of animals indicated in parentheses. For each variable, full results of repeated measures (RM) ANOVA are reported in Table 1. **Denotes p < 0.01. **(A)** At P21, there were no differences between the genotypes in sleep duration in light and dark phases. **(B)** At P70, the genotype × phase interaction was statistically significant (*p* < 0.001). *Post hoc t*-tests indicate that *Fmr1* KO (*n* = 19) animals had less sleep than controls (*n* = 19) in the light phase (*p* = 0.005). **(C)** At P180, the genotype × phase interaction was statistically significant (*p* = 0.015). *Post hoc t*-tests indicate that *Fmr1* KO animals (*n* = 21) than controls (*n* = 21) in the light phase (*p* = 0.004).

We assessed sleep in juvenile control (*n* = 19) and *Fmr1* KO (*n* = 23) mice at P21. At this age, neither the main effect of genotype nor the genotype × phase interaction were statistically significant (Table 1). Mean percent times asleep were similar for both genotypes in both phases (Figure 2A).

**Table 1:** *Post hoc* ANOVA results of average sleep times across Days 2–6 for the models presented.

Model/Age	Interaction	Main effect	*F*_(df,error)_ value	*P*-value
*Fmr1* KO P21	Genotype × Phase		*F*_(1,40)_ = 2.279	0.139
		Phase	*F*_(1,40)_ = 415.703	<0.001*
		Genotype	*F*_(1,40)_ = 0.037	0.848
*Fmr1* KO P70	Genotype × Phase		*F*_(1,36)_ = 11.687	0.002*
		Phase	*F*_(1,36)_ = 778.209	<0.001*
		Genotype	*F*_(1,36)_ = 2.204	0.146
*Fmr1* KO P180	Genotype × Phase		*F*_(1,40)_ = 6.498	0.015*
		Phase	*F*_(1,40)_ = 421.000	<0.001*
		Genotype	*F*_(1,40)_ = 4.090	0.05*
*Fmr1/Fxr2* P70	Genotype × Phase		*F*_(1,61)_ = 11.959	0.001*
		Phase	*F*_(1,61)_ = 350.108	<0.001*
		Genotype	*F*_(1,61)_ = 8.807	0.004*

The genotype × phase interaction as well as main effects of phase and genotype are presented with the corresponding F values and p-values. Statistically significant results are indicated with a “*”.

At P70 (young adult; Figure 2B), the phase × genotype interaction was statistically significant (*p* < 0.001; Table 1). *Post hoc t*-tests indicate that *Fmr1* KO mice (*n* = 19) had significantly (*p* = 0.007) reduced sleep only in the light phase compared to controls (*n* = 19). Mean differences were 6.39%.

At P180 (adult), the phase × genotype interaction was statistically significant (*p* = 0.015; Table 1; Figure 2C). *Post hoc t*-tests indicate that *Fmr1* KO mice (*n* = 21) slept less than controls (*n* = 21) in the light phase only (*p* = 0.004). Mean differences were 8.0%.

We asked if the sleep deficits in *Fmr1* KO mice at P180 could be reversed by treatment with R-baclofen, a GABA_B_ agonist. R-baclofen treatment reverses other behavioral and physiological phenotypes in adult *Fmr1* KO mice (Henderson et al., 2012; Qin et al., 2015a). We administered R-baclofen by daily i.p. injections (1.5 mg/kg). We used a within subjects’ design, i.e., sleep behavior was monitored in mice during daily i.p. injections of normal saline for 3 days followed by 2 days of daily R-baclofen i.p. injections. Prior to sleep monitoring, mice were acclimated to daily i.p. injections of saline for 6 days and 1 day of acclimation to the home-cage monitoring system. The sleep deficit in the light phase was not reversed by treatment with R-baclofen (57.7% sleep in the light phase during saline injections compared to 58.7% sleep during R-baclofen injections; *p* = 0.67, paired *t*-test). Moreover, R-baclofen did not affect sleep duration in the dark phase.

### Sleep Deficiencies in *Fmr1*/*Fxr2* Mice: Effects of Additional *Fxr* Deletion

**Figure 3:**
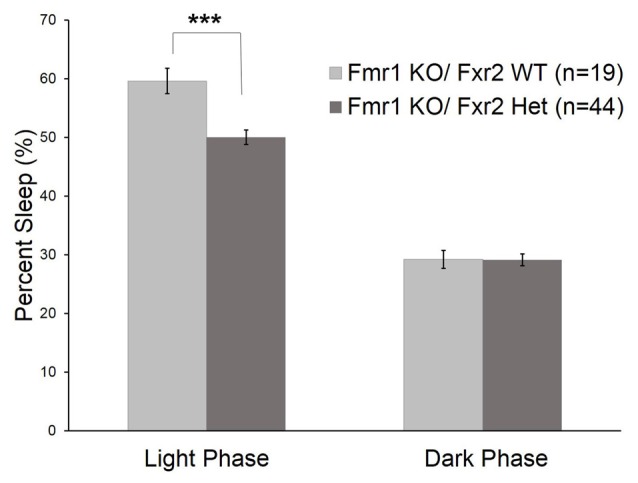
Sleep duration in *Fmr1* KO/*Fxr2* WT (*n* = 19) and *Fmr1* KO/*Fxr2* Heterozygous (Het; *n* = 44) mice at P70. Bars are the means ± SEM of sleep averaged across Days 2–3 on the number of animals indicated in parentheses. Full results of RM ANOVA are reported in Table 1. The genotype × phase interaction was statistically significant (*p* = 0.001), indicating that total sleep time in *Fmr1* KO/*Fxr2* Het animals was reduced compared with *Fmr1* KO/*Fxr2* WT animals in the light phase (*p* < 0.001). ***p < 0.001.

We asked if the *Fxr2* paralog was involved in sleep regulation in *Fmr1* KO mice. The absence of *Fxr2* in *Fmr1* KO mice exacerbates circadian rhythm abnormalities (Zhang et al., 2008). To see if this role of *Fxr2* in circadian rhythm extends to sleep, we studied *Fmr1* KO mice with (*Fmr1* KO/*Fxr2* WT) or haploinsufficient (*Fmr1* KO/*Fxr2* Het) for *Fxr2* at P70. We found that the phase × genotype interaction was statistically significant (*p* = 0.001; Table 1). In the light phase, *Fmr1* KO/*Fxr2* Het animals slept significantly less than *Fmr1* KO/*Fxr2* WT animals (*p* < 0.001; mean difference of 10%), but in the dark phase percent times were similar for both genotypes (Figure 3).

## Discussion

The results of our study show that *Fmr1* expression plays a role in the regulation of sleep physiology, and that its influence becomes apparent in adulthood. Additionally, *Fxr2*, an *Fmr1* paralog, appears to have a further influence on sleep physiology in mice. Our data highlight sleep physiology as an important phenotype in FXS that needs further characterization in patients. Moreover, abundant data support the importance of sleep in behavior and brain function (Picchioni et al., 2014; Kreutzmann et al., 2015; Saré et al., 2016a). Accordingly, correction of sleep abnormalities in FXS patients offers a promising therapeutic strategy. The effects of such therapies on sleep and ultimately on behavioral outcomes can be tested in FXS mouse models.

There are several strengths to our current studies, as well as a few limitations. First, we conducted a cross-sectional study to investigate sleep across development. We had good statistical power for our analysis. We controlled for several of the variables problematic in human clinical studies. Our animals were well matched for age; we used the same measure of sleep across all studies; all animals had not had any previous exposure to drugs. However, because of the nature of our study, we only have information about total sleep time, and we cannot measure sleep stages or sleep bout duration, which could inform us whether sleep fragmentation was occurring. For these questions, an electroencephalogram (EEG) study would be informative.

Hyperactivity is one of the common phenotypes detected in the *Fmr1* KO mouse model in both the active (Saré et al., 2016b) and inactive phases (Liu et al., 2011). Although hyperactivity and reduced sleep might be mediated by a similar mechanism, it is important to note that we are not detecting hyperactivity, *per se*. First, hyperactivity is traditionally assessed in a novel open-field environment larger than a typical mouse home-cage, whereas we are assessing sleep in the home-cage. Both tests use beam breaks to detect movement of the animal, but the criteria are different. A continuous measure of the number of beam breaks is used to measure activity in the open field. In the home-cage monitoring system, an animal is considered awake if it breaks a single beam in a 40 s epoch or if it breaks numerous beams in a 40 s epoch. Hyperactivity and decreased sleep duration may go hand in hand, but it is also possible that a hyperactive animal has the same number or even fewer awake epochs than a more sedentary animal.

Our finding of decreased sleep time in the light phase in *Fmr1* KO mice contrasts with results in the *dfmr1* model, in which sleep duration was increased (Inoue et al., 2002; Bushey et al., 2009). These phenotypic differences could reflect the absence of both *Fmr1* paralogs, *Fxr1* and *Fxr2*, in flies. In our study, both *Fmr1* KO and *Fmr1* KO* /Fxr2* Het mice had decreased sleep time in the light phase. The effects of loss of *Fxr1* in mice could not be tested due to the poor viability of *Fxr1* KO mice (Mientjes et al., 2004). Reduced sleep duration only in the light phase (the animal’s inactive phase) suggests that the sleep effect is modulated by circadian rhythms, and that sleep deficiency and circadian rhythm disruption are linked in *Fmr1* KO animals. These results align with the circadian rhythm disruption previously reported in *Fmr1* KO animals (Zhang et al., 2008).

Although we did not detect sleep deficiencies in *Fmr1* KO mice at P21 by activity monitoring, there is electrophysiological and calcium imaging evidence that cortical neuronal firing and synchrony during sleep are abnormally high in *Fmr1* KO mice at P14–P16 suggesting that Up/Down states are not normal (Gonçalves et al., 2013). How this may progress into circadian alterations and reduced sleep in the light phase is unknown. However, circadian rhythm in P21 animals is not as defined as in adult animals (Hagenauer et al., 2009), which may mask potential differences between genotypes. Additionally, at P21, the mouse brain is still developing, and is thought to be somewhat equivalent to a human of around 3 years old. By P70, the mouse brain is mature and equivalent to about a 20 year old human (Semple et al., 2013). It is during this period from P21 to P70 that *Fmr1* KO mice develop a statistically significant genotype × phase interaction, suggestive of a circadian rhythm disruption, implying that this abnormality unfolds during brain maturation. It is interesting to note that most behavioral abnormalities reported in *Fmr1* KO mice have been assessed at 2 months of age and later (Yan et al., 2004; Spencer et al., 2005; Zhao et al., 2005; Moon et al., 2006; Liu and Smith, 2009; Liu et al., 2011; Ding et al., 2014; Qin et al., 2015a,b), suggesting that sleep/circadian rhythm problems may develop before other behavioral impairments. The timing of the development of sleep abnormalities in the mouse may inform the timing of screening for sleep problems in FXS children. It also may help to determine the best window for treatment.

Although the differences in total sleep time between *Fmr1* KO and control mice are relatively small (6% in the light phase in P70 animals and 8% in the light phase in P180 animals), these differences may very well be biologically significant. Sleep has an important role in brain development and plasticity (Picchioni et al., 2014; Kreutzmann et al., 2015). Studies of chronic partial sleep loss have revealed that deficits are similar to those observed in acute total sleep deprivation. These deficits were in areas of cognition and neurobehavioral function (Van Dongen et al., 2003). Chronic sleep restriction in mice leads to long-lasting effects on behavior, even after restoration of normal sleep (Saré et al., 2016a). These behavioral changes could be mediated by changes in plasticity in the brain that are not recovered during subsequent sleep periods. Studies have shown that cellular processes implicated in plasticity such as myelination, cellular stress and neurogenesis are affected by sleep restriction and may not recover even after regaining sleep (Tung et al., 2005; Picchioni et al., 2014; Kreutzmann et al., 2015). The consequences of chronically reduced sleep in the light phase in FXS may be an important contributor to the brain and behavioral manifestations of the disorder.

Given the potential impact of sleep deficits in the unfolding of fragile X phenotypes, it may be important to determine the mechanisms by which sleep is dysregulated in the *Fmr1* KO mice. There are two processes controlling the drive to sleep. One is by means of the circadian clock and the other is a homeostatic drive (Borbély and Achermann, 1999). In their study of circadian rhythm, Zhang et al. examined the expression of clock genes involved in circadian rhythm in both *Fmr1* KO and *Fmr1/Fxr2* double Het animals. They found that both models showed rhythmicity in the clock genes in the superchiasmatic nucleus (SCN). However, *Fmr1* KO/*Fxr2* Het animals did show increased expression of *Cry1* at the beginning of the active phase (Zhang et al., 2008). Given that both *Fmr1* KO and *Fmr1* KO/*Fxr2* Het animals show decreased sleep, this mechanism is unlikely to account for the change in sleep duration. Downstream of the SCN, in the liver which is noted as the peripheral clock, regulation of *Bmal1*, *mPer1*, *mPer2* and *Npas2* in the *Fmr1/Fxr2* Het animals was altered relative to controls (Zhang et al., 2008). Again, as these changes did not occur in *Fmr1* KO mice, they cannot fully explain the reduced sleep phenotype. Both *Fmr1* KO and *Fmr1/Fxr2* Het animals did have increased expression of *Cry1* in the liver at the beginning of the active phase (Zhang et al., 2008), so it is possible that *Cry1* regulation may contribute to the reduced sleep in both *Fmr1* KO and *Fmr1* KO/*Fxr2* Het animals.

The other process controlling sleep is the homeostatic drive. Understanding the molecular mechanisms that manage the homeostatic regulation of sleep in *Fmr1* KO mice is much more difficult because the process is less understood. One mechanism of sleep initiation, particularly nonREM sleep, is activation of GABA receptors (Lancel, 1999). It has been shown that *Fmr1* KO mice have downregulation of both GABA_A_ and GABA_B_ receptors (Pacey et al., 2011; Liu et al., 2013). In our study, R-baclofen, a GABA_B_ agonist, did not improve sleep in adult *Fmr1* KO mice. We controlled for effects of i.p. injections and acclimation to the monitoring system on sleep duration. We only tested mice at 6 months of age, and it is possible that the treatment might be effective in younger mice. Based on our results, we think it unlikely that GABA_B_ receptors are involved in the sleep deficits observed in *Fmr1* KO and *Fmr1* KO/*Fxr2* Het animals. Future work will address the role of GABA_A_ receptors in mediating the sleep deficits in *Fmr1* KO and *Fmr1* KO/*Fxr2* Het animals.

Our findings highlight the importance of *Fmr1* and *Fxr2* in the regulation of sleep in adult mice. With loss of *Fmr1* (either alone or in combination with *Fxr2*), adult animals have reduced total sleep time in the light phase. Our data in conjunction with clinical reports (Musumeci et al., 1995; Gould et al., 2000) suggest that patients without *FMR1* expression are likely to have chronically reduced night-time sleep. Our findings in *Fmr1* KO mice suggest that sleep problems (such as reduced sleep) should be more thoroughly examined in FXS patients and considered as targets for therapeutic intervention. Additionally, these studies show that *Fmr1* KO mice and *Fmr1* KO/*Fxr2* Het mice may be useful for further examining the consequences and potential treatments for the sleep problems in FXS.

## Author Contributions

RMS designed the study, performed experiments, analyzed data, interpreted the data and drafted the manuscript; LH performed experiments; ML performed experiments and drafted the manuscript; AT performed experiments; CAS performed experiments; CBS designed the study, interpreted the data, and drafted the manuscript. All authors read and approved the final version of the manuscript.

## Conflict of Interest Statement

The authors declare that the research was conducted in the absence of any commercial or financial relationships that could be construed as a potential conflict of interest.

## Abbreviations

CLAMS: comprehensive laboratory animal monitoring system
FXS: Fragile X syndrome
SEM: standard error of the mean.
